# MicroRNA manipulation in colorectal cancer cells: from laboratory to clinical application

**DOI:** 10.1186/1479-5876-10-128

**Published:** 2012-06-20

**Authors:** Muhammad Imran Aslam, Maleene Patel, Baljit Singh, John Stuart Jameson, James Howard Pringle

**Affiliations:** 1 Department of Cancer Studies and Molecular Medicine, University of Leicester, Robert Kilpatrick Clinical Sciences Building, Leicester Royal Infirmary, P O Box 65, Leicester, LE2 7LX, United Kingdom; 2 Department of Colorectal Surgery, University Hospitals of Leicester Leicester NHS Trusts, Leicester General Hospital, Gwendolen Road, Leicester, LE5 4PW, United Kingdom

**Keywords:** Antisense, CRC, Oligonucleotide, LNA, MicroRNA, miRNA-sponges, miR-mask

## Abstract

The development of Colorectal Cancer (CRC) follows a sequential progression from adenoma to the carcinoma. Therefore, opportunities exist to interfere with the natural course of disease development and progression. Dysregulation of microRNAs (miRNAs) in cancer cells indirectly results in higher levels of messenger RNA (mRNA) specific to tumour promoter genes or tumour suppressor genes. This narrative review aims to provide a comprehensive review of the literature about the manipulation of oncogenic or tumour suppressor miRNAs in colorectal cancer cells for the purpose of development of anticancer therapies. A literature search identified studies describing manipulation of miRNAs in colorectal cancer cells in vivo and in vitro. Studies were also included to provide an update on the role of miRNAs in CRC development, progression and diagnosis. Strategy based on restoration of silenced miRNAs or inhibition of over expressed miRNAs has opened a new area of research in cancer therapy. In this review article different techniques for miRNA manipulation are reviewed and their utility for colorectal cancer therapy has been discussed in detail. Restoration of normal equilibrium for cancer related miRNAs can result in inhibition of tumour growth, apoptosis, blocking of invasion, angiogenesis and metastasis. Furthermore, drug resistant cancer cells can be turned into drug sensitive cells on alteration of specific miRNAs in cancer cells. MiRNA modulation in cancer cells holds great potential to replace current anticancer therapies. However, further work is needed on tissue specific delivery systems and strategies to avoid side effects.

## Background

Colorectal cancer (CRC) is the third most common neoplasm worldwide. According to the International Agency for Research on Cancer, approximately 1.24 million new cases of CRC were detected worldwide in 2008 [1]. The incidence of CRC is on the rise in developing countries, southern and eastern Europe [2-4]. Contrary to the current trend in Europe, the incidence of CRC in the USA has fallen in the last two decades [5]. The lifetime risk for developing CRC [6] in men is 1 in 16 whereas in women it is 1 in 20 (National Statistics, UK). The development of CRC follows the sequential progression from adenoma to carcinoma [7]. The initial genetic alteration results in adenoma formation in which cells exhibit autonomous growth. During the further course of carcinogenesis, intestinal epithelial cells acquire the characteristics of invasion and potential for metastasis. Therefore opportunities exist to interfere with the natural course of disease development and improve cancer specific survival. Such therapeutic interferences can potentially be chemo preventive for high risk individuals, the early detection of colorectal neoplasia, chemotherapies to down stage the surgically resected or resectable cancers, and therapies for palliation of symptoms in advanced stage cancer. Discovery of microRNAs (miRNAs) and their utility in RNA interference has opened a new era of cancer research and potential of new therapies for cancer treatment. This narrative review aims to provide a comprehensive review of the literature about the manipulation of miRNAs in colorectal cancer cells and tissue for the purpose of development of anticancer therapies. Principles of miRNA manipulation and common methods of modulation in vitro and vivo are discussed in detail for the general understanding of readers. This review also aims to provide the update on the role of miRNAs in CRC development and the diagnostic utility of circulating miRNAs

## Methods

A search was performed using Medline, PubMed and The Cochrane Library databases from 2000 to 2011 to identify articles reporting the role of miRNAs in colorectal cancer development, diagnosis and therapy. The following MeSH search headings were used: ‘microRNA’ and ‘colorectal cancer’. The search was further extended by using the following text words and their combinations: ‘microRNA’, ‘blood’, ‘circulation’, ‘diagnosis’, ‘screening’, ‘therapy’, ‘manipulation’, ‘modulation’, ‘stem cells’ and ‘miRNAs’. ‘The related articles’ function in PubMed was used to broaden the search. All the abstracts, studies and citations found were reviewed. The most recent date of the search was 16 July 2011. Information about colorectal cancer related miRNAs was extracted on the following areas: cancer development & progression; diagnostic utility; manipulation in vitro and vivo; development of therapies. Detailed information was extracted from studies that met the inclusion criteria: Studies conducted on human and non-human cells or tissues; blood-based miRNAs- in colorectal cancers and studies published in the English literature. Because of the lack of randomized controlled trials and the heterogeneous nature of the available data, no attempt was made to perform quantitative meta-analyses. In the absence of standard criteria for the quality assessment of laboratory-based, observational studies on miRNA and heterogeneity of outcome measures included in this narrative review, no quality assessment of included studies was carried out. As the study is only a narrative review, no ethical permission or approval was required.

## Results

The literature search identified 48 original scientific studies and review articles in which some or all of the outcomes of interest were reported. 18 additional articles and web-based information sites were selected to provide a general background to miRNA and colorectal neoplasia. 36 additional studies were included to supplement the information in colorectal cancer development, detection of circulating miRNAs, and principles of miRNA therapy, design and delivery. This article provides a comprehensive review of the different approaches for restoration of tumour suppressor miRNAs expression and methods of knocking down the tumour promoter miRNAs in cancer cells. Not all the methodologies of miRNA manipulation have been applied to CRC cells and some have been experimented only on other solid organ cancers. As description and elaboration of these methods might provide an insight to future CRC therapies designed on miRNA manipulations, the authors have included principle studies focussing on mechanisms of such manipulation.

## Discussion

### MiRNA biogenesis and function

**Figure 1:**
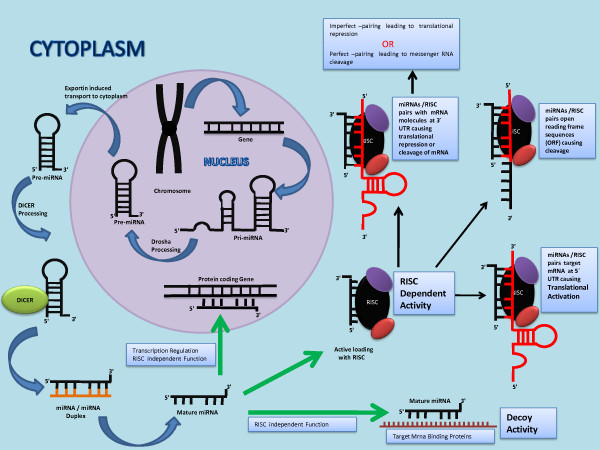
**Figure illustrates the biogenesis of miRNAs in the cellular nucleous, its transport to the cytoplasm, and processing by Drosha and Dicer Enzymes.** The figure also illustrates the RISC incorporation of miRNAs for functional activity in different pathways of translational inhibition or activation. In brief, miRNAs are mostly transcribed from intragenic or intergenic regions by RNA polymerase II into primary transcripts (pri-miRNAs) of variable length (1 kb- 3 kb). In the nucleus Pri-miRNA transcript is further processed by the nuclear ribo-nuclease enzyme ‘Drosha’ thereby resulting in a hairpin intermediate of about 70–100 nucleotides, called pre-miRNA. The pre-miRNA is then transported out of the nucleus by a transporting protein exportin-5. In the cytoplasm, the pre-miRNA is once again processed by another ribonuclease enzyme ‘Dicer’ into a mature double-stranded miRNA. Two strands of double stranded miRNA (miRNA/miRNA* complex) are separated by Dicer processing. After strand separation, mature miRNA strand (miRNA- also called the guide strand) is incorporated into the RNA-induced silencing complex (RISC), whereas the passenger strand, denoted with a star (miRNA*) is commonly degraded. This miRNA/RISC complex is responsible for miRNA function. If on miRNA cloning, or miRNA array, the passenger strand is found at low frequency (less than 15% of the guide strand) it is named miR*. However, if both passenger and guide strand are equal in distribution, then these two strands are named the 3p and 5p version of miRNA depending on their location to either 5' or 3' of the miRNA molecule. In this case both strands can potentially incorporate in position into the RISC complex and have a biological role [9-14]. The specificity of miRNA targeting is defined by Watson–Crick complementarities between positions 2 to 8 from the 5 primed end of the miRNA sequence with the 3′ untranslated region (UTR) of their target mRNAs. When miRNA and its target mRNA sequence show perfect complementarities, the RISC induces mRNA degradation. Should an imperfect miRNA–mRNA target pairing occur, translation into a protein is blocked [9,10]. Regardless of which of these two events occur, the net result is a decrease in the amount of protein encoded by the mRNA targets. Each miRNA has the potential to target a large number of genes (on average about 500 for each miRNA family). Conversely, an estimated 60% of the mRNAs have one or more evolutionarily conserved sequence that is predicted to interact with miRNAs [9,10,15]. miRNAs have been shown to bind to the open reading frame or to the 5′ UTR of the target genes and, in some cases, they have been shown to activate rather than to inhibit gene expression [16]. Researchers have also reported that miRNAs can bind to ribonucleoproteins in a seed sequence and a RISC-independent manner and then interfere with their RNA binding functions (decoy activity) [17]. MiRNAs can also regulate gene expression at the transcriptional level by binding directly to the DNA [18] .

miRNAs are single-stranded, evolutionarily conserved, small (17–25 ribonucleotides) noncoding [8] RNA molecules. MiRNAs function as negative regulators of target genes by directing specific messenger RNA cleavage or translational inhibition through RNA induced silencing complex termed as RISC [9,10]. So far around fourteen hundred mature human miRNAs have been described in the Sanger miRBase version 17 (An international registry and database for miRNAs nomenclature, targets, functions and their implications in different diseases). Figure 1 illustrates the biogenesis and mechanism of action of miRNAs [11-18].

### MiRNAs and colorectal cancer carcinogenesis

**Figure 2:**
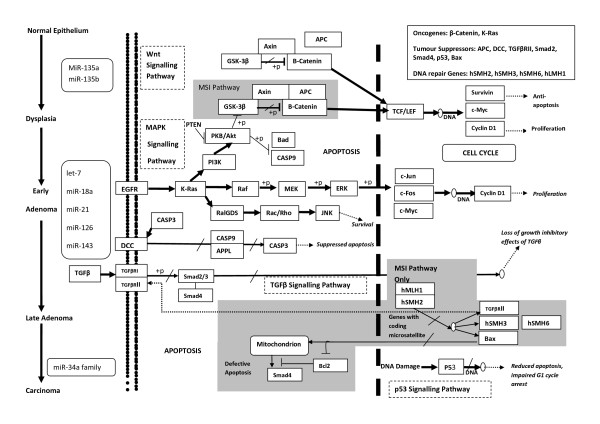
**Carcinogenesis of colorectal cancer cells and role of different miRNAs in cancer pathways.** In the carcinogenesis of CRC, higher levels of miR-135a & miR-135b are associated with low levels of Adenomatous Polyposis Coli (APC) , which in turn leads to activation of the Wnt signalling pathway. Activation of the Wnt signalling pathway is a major tumour initiating event in the colonic epithelium. The low level of APC associated β-catenin degradation complex results in the formation of free cytoplasmic β-catenin that enters the nucleus and activates Wnt-regulated genes through its interaction with TCF (T-cell factor) family transcription factors and concomitant recruitment of coactivators (Survivin, c-Myc and Cyclin D1). As a consequence, there is a lack of apoptosis and increase proliferation of abnormal cells that results in autonomous growth and formation of adenoma. During the course of carcinogenesis, cells in adenoma accumulate few other genetic alterations leading to activation of other signalling pathways e.g. mitogen activated protein kinase (MAPK), Phosphatidylinositol 3-kinases (PI3K) and transforming growth factor-beta (TGFβ) pathways. The let-7 miRNA family, miR-18a* and miR-143 are adept at inhibiting the KRAS translation hence switching “off” the MAPK phosphorylation and inactivation of downstream transcription factors c-Myc, c-Fos and c-Jun. Furthermore, a targeted degradation of PTEN and p85β by miR-21 and miR-126 respectively , blocks the PI3K-Akt pathway. These changes drive the early adenoma to a large advanced adenoma. The loss of p53 function is associated with low expression levels of miR-34a family, indicating the role of this miRNA family in the transformation of adenoma to the carcinoma.

MiRNAs play an important role in colorectal tumour biology including; oncogenesis; progression; invasion; metastasis and angiogenesis [19-22]. Initiation and progression of colorectal neoplasia results from sequential accumulation of genetic alterations in oncogenic and tumour suppressor genes in colonic epithelium [23]. MiRNAs interfere with these genetic mutations and are involved in different stages of cancer of colorectal neoplasia. Slaby and colleagues summarized the role of different miRNAs in the development of colorectal cancer and emphasized the importance of Adenomatous Polyposis Coli (APC), Tumour Protein 53 (TP53) gene mutations and the WNT signalling Pathway [24]. The initiation of colonic neoplasia is strongly linked to inactivation of the APC gene and activation of the WNT Signalling Pathway. APC inactivation has been found in more than 60% of colonic tumours and such inactivation is associated with up regulation of miR-135a/b in colonic epithelial cells [23,25,26]. Accumulation of any further somatic mutations leads to further dysregulation of miRNAs and activation of additional downstream pathways. For example let-7, miR-18a* & miR-143 are strongly linked to KRAS knockdown and activation of the EGFR-MAPK pathway [27-29] whereas miR-21 and miR-126 are associated with augmentation or inactivation of the phosphatidylinositol-3-kinase (PI-3-K) pathway repectively [30,31]. Activation of these downstream pathways results in autonomous tumour cell growth, increased cell survival, and initiation of angiogenesis. Loss of P53 is a critical step in transformation of adenoma to adenocarcinoma as nearly 50–70% of colonic adenocarcinomas are found to be P53 mutant [23]. miR-34a has been identified as a direct downstream target of P53 and the replacement of miR-34a has achieved p53 induced effects of apoptosis and cell cycle arrest [32]. A commonly up regulated miR-17-92 cluster (miR-17, miR-18a, miR-19a, miR-20a, miR-19b & miR-92a) also drives the progression of adenoma to adenocarcinoma by up regulation of c-myc [33]. Figure 2 summarises the interaction of different miRNAs in signalling pathways for colorectal cancer development and progression.In the KEGG pathway, it shows the interaction of different miRNAs in the formation of adenoma and its progression to adenocarcinoma.

Another cancer pathway- the second serrated neoplasia pathway has recently gained acceptance and is for the most part, APC and TP53 mutation independent. It involves distinct molecular features of somatic BRAF mutation concordance with high CpG islands methylation phenotype (CIMP-H) and microsatellite instability (MSI+) associated with mutt homologue 1 (MLH1) methylation [34,35]. Involvement of the miRNAs in the latter pathway is slowly emerging and would require further functional studies to find the link of miRNAs associated with this pathway.

**Figure 3:**
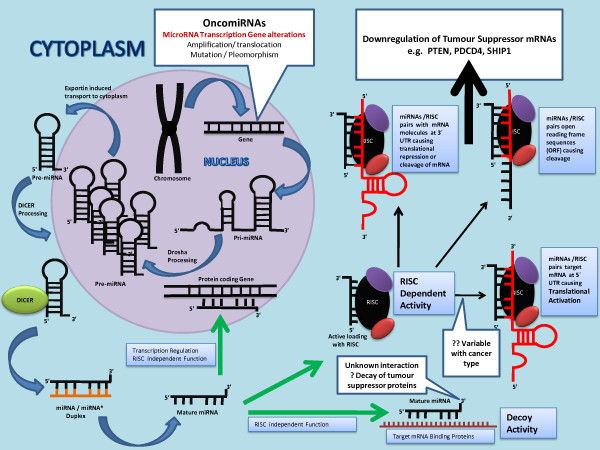
**Figure illustrates the mechanism of biogenesis and function of OncomiRNAs.** Amplification, translocation, mutation or pleomorphism in miRNA transcribing genes results in over production of pri-miRNA and pre-miRNAs in the nucleous. Further processing by the DICER results in higher levels of mature miRNAs in the cytoplasm. These overexpressed miRNAs target tumour suppressor mRNAs in the cytoplasm and lead to the downregulation of mRNAs.

**Figure 4:**
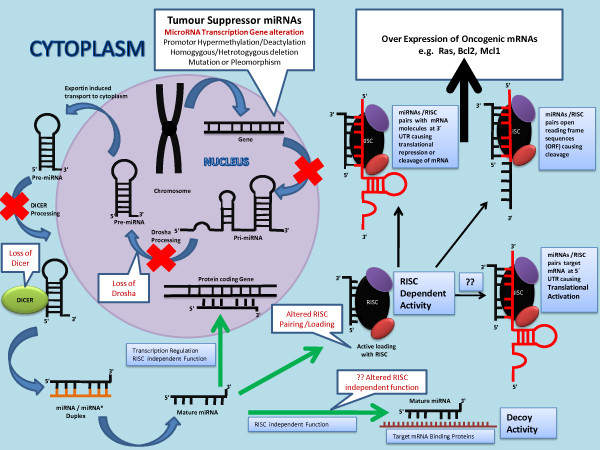
**Figure shows the proposed mechanism of biogenesis and function of tumour suppressor miRNAs.** Promotor hypermethylation/deactylation, homogygous/hetrotogygous deletion, mutation or pleomorphism in miRNA transcription gene results in under production or complete loss of pri-miRNAs. Defects in miRNA processing machinery i.e. ineffective processing by Drosha/Dicer or defective pairing with RISC can result into inefficient levels of mature miRNAs in the cytoplasm. Low levels of tumour suppressor miRNAs result in over expression of oncogenic mRNAs (Ras, Bcl2, Mcl1).

Functional and mechanistic studies of miRNAs have shown that the replacement or knockdown of distinct miRNAs in vitro resulted in distinct cytogenetic abnormalities leading to either tumour cell proliferation or apoptosis [20]. That’s why it is believed that dysregulation of miRNA genes that target mRNAs for tumour suppressor or oncogenes contributes to tumour formation by inducing cell proliferation, invasion, angiogenesis and decreased cell death [19]. This has further lead to belief that over expressed miRNAs in tumour cells function by inhibiting different tumour suppressor genes whereas miRNAs that are often found silenced in tumour cells downregulate the expression of oncogenes in normal tissue (Figures 3 &4). Amplification, translocation, pleomorphism or mutation in miRNA transcribing genes results in over production of miRNAs. In contrast, mutation, deletion, promoter methylation or any abnormalities in the miRNA biogenesis results in silencing of miRNAs in tumour cells [19]. Table 1 summarizes commonly expressed miRNAs in colorectal cancer tissue in comparison to adjacent healthy colonic mucosa [36-49].

**Table 1:** Summary of dysregulated miRNAs in CRC compared to adjacent normal mucosa

**Studies**	**Downredulated miRNAs in CRC tissue**	**Upregulated miRNAs in CRC tissue**
**Michael, et al, 2003**[36]	let-7, **miR-16**, miR-24, miR-26a, miR-102, miR-143, miR-145, miR-200b	
**Volinia, et al, 2006**[37]	let-7a-1, miR-9-3, miR-23b, miR-138, miR-218	**miR-16**, miR-17-5p, miR-20a, miR-21, miR-29b ,miR-141, **miR-195**, miR-199a
**Xi , et al, 2006**[32]	let-7b, **let-7 g** , miR-26a , miR-30a-3p, **miR-132**, miR-181a, **miR-181b**, miR-296, miR-320, miR-372	miR-10a, miR-15b ,miR-23a, miR-25, miR-27a, miR-27b, ** miR-30c **, miR-107, **miR-125a**, miR-191, miR-200c, miR-339
**Bandrés, et al, 2006**[38]	miR-133b, miR-145	** miR-31 **, miR-96, miR-135b, miR-183
**Akao, et al, 2006**[27,39]	miR-143, miR-145, let7	
**Nakajima, et al,2006**[40]		**let-7 g, miR-181b **, miR-200c
**Lanza, et al, 2007**[41]		miR-17-5p, miR-20, miR-25, miR-92, miR-93-1, miR-106a
**Rossi, et al, 2007**[42]	miR-200b, miR-210 , miR-224	miR-19a, miR-20, miR-21, miR-23a, miR-25, miR-27a, miR-27b, miR-29a, miR-30e, miR-124b, **miR-132**, **miR-133a**, miR-135b, miR-141, miR-147, miR-151, miR-152, miR-182, miR-185
**Monz, et al, 2008**[43]	miR-145	miR-17-5p ,miR-21, ** miR-30c **, miR-106a, miR-107, miR-191, miR-221
**Schepeler, et al, 2008**[44]	miR-101, miR-145, miR-455, miR-484	miR-20a, miR-92, miR-510, miR-513
**Schetter, et al, 2008**[45]		miR-20a, miR-21, ** miR-106a **, ** miR-181b ,** miR-203
**Arndt, et al, 2009**[46]	miR-1, miR-10b, miR-30a-3p, miR-30a-5p, **miR-30c, miR-125a**, **miR-133a,** miR-139, miR-143, miR-145, **miR-195**, miR-378*, miR-422a, miR-422b, miR-497	miR-17-5p, miR-18a, miR-19a, miR-19b, miR-20a, miR-21, miR-25, miR-29a, miR-29b, ** miR-31 **, miR-34a, miR-93, miR-95, miR-96, ** miR-106a **, miR-106b, miR-130b, ** miR-181b **, miR-182, miR-183, miR-203, **miR-224**
**Slattery, et al, 2011**[47]	miR-143, miR-145, miR-192, miR-215	miR-21, miR-21*, miR-183, miR-92a, miR-17, miR-18a, miR-19a, miR-34a

*Over expressed or under expressed miRNAs identified by two or more than two studies are underlined whereas the miRNAs with conflicting expression levels in different studies are identified in Bold.

### Diagnostic utility of circulating miRNAs

Recent studies have shown that tumour-derived miRNAs are present in human body fluids in a remarkably stable form and are protected from endogenous ribonuclease activity. In three different studies researchers have evaluated the suitability of circulating miRNAs as a diagnostic biomarker for CRC [50]. Preliminary studies suggest that CRC derived miRNAs are present in the circulation at detectable levels [51-54] and can accurately distinguish healthy controls from patients with CRC (Table 2). Significantly high sensitivity and specificity for detection of CRC holds promise for the use of circulating miRNAs as a diagnostic biomarker for CRC. Furthermore, the ability of a miRNA based blood assay to detect colonic adenoma can lead to its use in early detection and bowel cancer screening. Although a small number of studies have identified circulating miRNAs in CRC patients, the clinical utility of this is still questionable. This is due to an overlapping miRNA expression with other solid organ cancers and benign colonic diseases, and variability of individual miRNA expression with tumour site & stage. It is possible that the utility of tumour tissue specific expression-signature/profile may prove more informative and accurate in future clinical studies. Furthermore, the discovery of exosome mediated transport of miRNAs into the circulation, has shifted the focus of miRNA studies towards the isolation of tissue specific circulating exosomes and their contained miRNAs [55].

**Table 2:** Summary of sensitivity and specificity of different diagnostic circulating miRNAs

**Tissue type**	**Studies**	**Participants**	**Target MiRNAs**	**Diagnostic accuracy**	
				**Sensitivity%**	**Specificity%**
Whole Plasma	Pu, et al, 2010 [51]	CRC (n = 103)	miR-221	86	41
		Controls (n = 37)			
Plasma	Cheng , et al, 2011 [52]	CRC I-IV (n = 102)	miR-141	66.7	80.8
		Controls (n = 48)			
RNA	Ng, et al, 2009 [53]	CRC (n = 90)	miR-17-3p	64	70
		Controls (n = 40)	miR-92	89	70
	Huang, et al, 2010 [54]	CRC (n = 100)	miR-29	69	89.1
		Adenomas* (n = 37)		62.2*	84.7*
			miR-92a	84	71
		Controls (n = 59)		64.9*	81.4*

*Adenoma cases.

### Principles of miRNA therapy

The fact that miRNAs regulate multiple genes in a molecular pathway, makes them excellent candidates for gene therapy. One of the most appealing properties of miRNAs as therapeutic agents is their ability to target multiple genes, frequently in the context of a network, making them extremely efficient in regulating distinct biological cell processes relevant to normal and malignant cell homeostasis. The rationale for using miRNAs as anticancer drugs is based on two major principles:

1. MiRNA expression is deregulated in cancer compared with normal tissues

2. Cancer phenotype can be changed by targeting miRNA expression

MiRNA based gene therapy is based on these two principles where manipulation of miRNA expression levels in cancer tissue results in inhibition of tumour growth, apoptosis, blocking of invasion, angiogenesis and metastasis. Restoration of the normal equilibrium for cancer related miRNA expression levels can result in growth retardation and reduced cell viability both in vivo and in vitro experiments. The major obstacle in gene therapy is the safe delivery to specific target tissue without side effects. Rapid degradation by body nucleases and poor cellular uptake owing to the unfavourable chemical structure of synthetic miRNAs have forced researchers to try chemical modifications of synthetic oligonucleotides as well as a more effective means of delivery. To overcome these delivery hurdles, viral and non-viral strategies have been developed. Restoration of tumour-suppressor miRNAs in cancer cells is usually achieved in vitro by using adenovirus-associated vectors (AAV). These vectors do not integrate into the genome and are eliminated efficiently with minimal toxicity. There are multiple AAV serotypes available, allowing for the efficient targeting of specific tissues including colorectal tissue. The ability of miRNAs to regulate several genes, does create potential problem in terms of side effects. This is a major concern in miRNA therapeutics as such interactions may lead to toxic phenotypes formation in targeted cells [56]. This has been approached by using nano-particles and tissue specific non viral vectors. However, the concentration dependent knockdown of non specific targets still remains an unresolved issue. This article provides a comprehensive review of the different approaches for restoration of tumour suppressor miRNAs expression and methods of knocking down the tumour promoter miRNAs in cancer cells. Not all the methodologies mentioned in this article have been applied to CRC cells, but all have been investigated as a therapy for other cancers. The description and elaboration of these methods may provide an insight to miRNA therapies in CRC.

### Blocking oncogenic MiRNAs using antisense oligonucleotides

**Figure 5:**
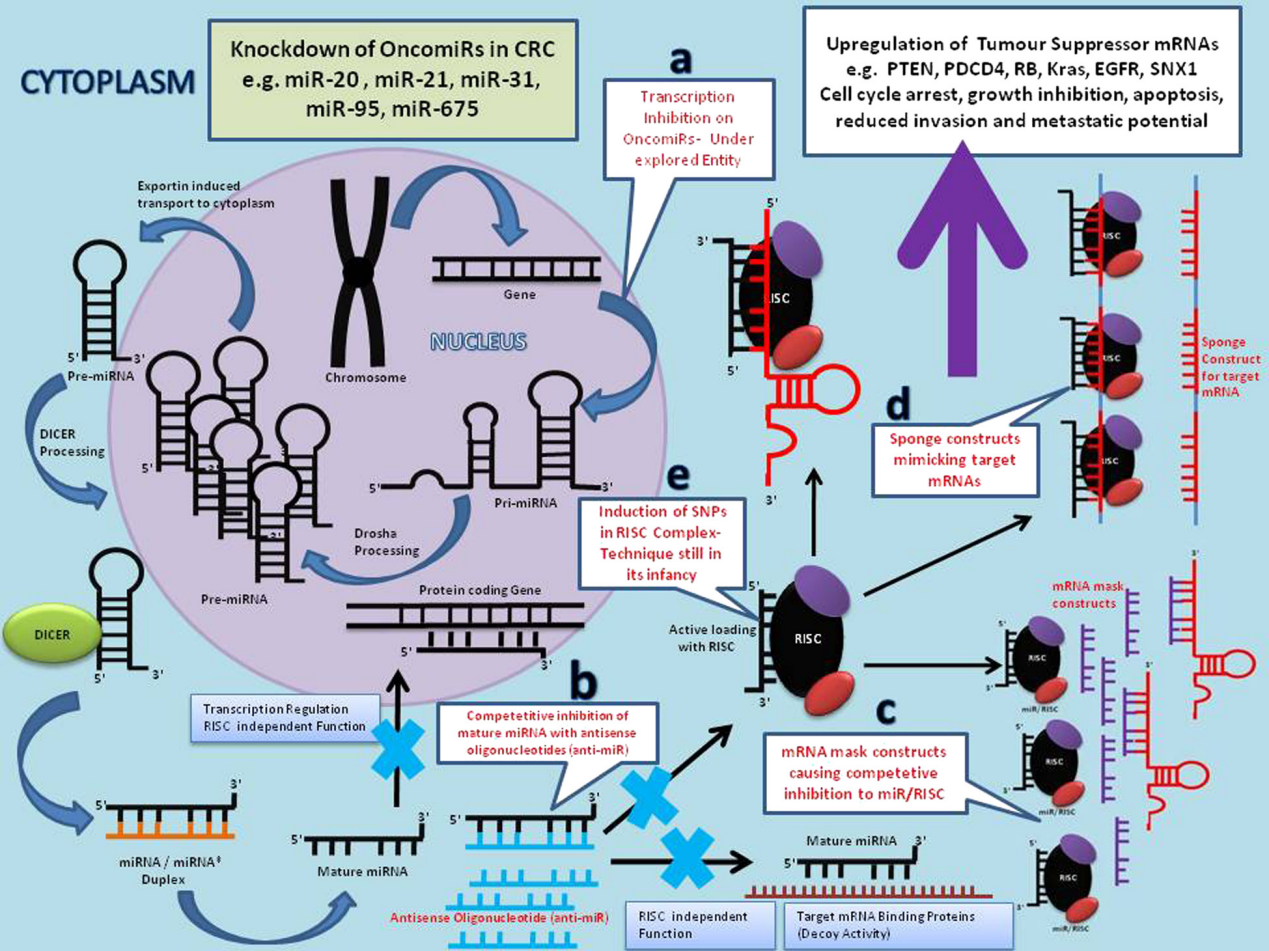
**Figure demonstrates the mechanism of Knockdown of oncomiRs.** The downregulation of tumour suppressor mRNAs can be reversed by a) transcriptional inhibition of genes transcribing miRNAs of oncogenic potential b) competitive inhibition of mature miRNAs with antisense oligonucleotides c) mRNA mask constructs causing competitive inhibition to miR/RISC d) sponge constructs mimicking target mRNAs e) induction of SNPs in RISC complex.

The demonstration that oncogenic miRNAs are upregulated in cancer provided a rationale to investigate the use of antisense oligonucleotides to block their expression. Antisense oligonucleotides work as competitive inhibitors of miRNAs, presumably by annealing to the mature miRNA guide strand and inducing degradation of mature miRNAs (Figure 5). The stability, and specificity for target miRNAs and the binding affinity of antisense oligonucleotides has been optimised by modifications to the chemical structure of the oligonucleotides [57]. In particular the introduction of 2′-O-methyl or 2′-O-methoxyethyl groups to oligonucleotides enhances resistance to nuclease enzyme and improves the binding affinities to RNA [58]. The silencing of endogenous miRNAs by this novel method has shown to be long lasting, specific and efficient both in vitro [59,60] and in vivo. In CRC cells lines, antimiR based blockage of oncogenic miRNAs (miR-20a, miR-21, miR-31, miR-95, miR-672) has not only shown to reduce cell proliferation, transformation and migration, but it has resulted in enhanced sensitivity to chemotherapy agents [61-66]. This strategy of sensitizing the chemotherapy resistant tumour cells with alteration of miRNA expression in tumour cells may result in improved response to traditional chemotherapy agents. Table 3 summarizes the studies manipulating the oncogenic miRNAs in colorectal cell lines.

**Table 3:** Summary of in vitro effects of antisense oligonucleotides and locked nucleic acid based oncogenic miRNA manipulation in colorectal cell lines

**miRNA**	**Cell line**	**Target**	**Method**	**Reported effects of miRNA manipulation**	**References**
**antisense oligonucleotides**					
miR-20a	SW480	BNIP2	anti–miR-20a	Increased sensitivity to fluorouracil, oxaliplatin, and teniposide	Chai, et al, 2011[61]
	SW620				
miR-21	RKO	Pdcd4	anti-miR-21	Reduced intravasation and distal metastasis	Asangani, et al, 2008[62]
miR-31	HCT-116		anti-miR-31	Reduced migration and increased invasion	Wang , et al, 2010[63]
				Reduced proliferation only with combined 5-FU	
miR-95	HCT-116 LoVo	SNX1	anti–miR-95	Reduces cell proliferation	Huang , et al, 2011[64]
		EGFR			
miR-675	CaCO2 HCT116	RB	anti-miR-675	Reduced cell growth and colony transformation	Tsang , et al, 2010 [65]
	HT-29 SW480				
**Locked nucleic acid (LNA)**					
miR-21	SW480	hMSH2 hMSH6	LNA anti-miR-21	Increased sensitivity to 5-FU	Valeri, et al, 2010 [67]
	HCT-116				
	RKO				

### Blocking oncogenic MiRNAs using locked nucleic acid (LNA) Constructs

LNA construct based anti-miR therapeutic strategy has been extensively explored by researchers studying the miRNA based antiviral therapy for chronic hepatitis-C [66]. LNA nucleosides are a class of nucleic acid analogues in which the ribose ring is 'locked' by a methylene bridge connecting the 2′-O atom and the 4′-C atom. By locking the molecule with the methylene bridge, LNA oligonucleotides display unprecedented hybridization affinity towards complementary single-stranded RNA and complementary single-stranded or double-stranded DNA [68]. In addition, they display excellent mismatch discrimination and high aqueous solubility. So-called 'LNA anti-miR' constructs has been used successfully by Valeri and colleagues [67] to knock down miR-21 in colon adenocarcinoma cell lines.

### Blocking oncogenic MiRNAs using MiRNA sponge constructs

miRNA sponges based techniques to manipulate oncogenic miRNAs is still in its infancy and has not been studied in detail in CRC cells. miRNA sponges are transcripts that contain multiple tandem-binding sites to a miRNA of interest and are transcribed from mammalian expression vectors. Ebert and colleagues recently reported the use of miRNA sponges in mammalian cells [69]. The authors reasoned that miRNA target sequences expressed at high levels could compete with bona fide targets in a cell for miRNA binding (Figure 5). To increase the affinity of these decoy transcripts, the researchers introduced not only multiple miRNA binding sites, but also a bulge at the position normally cleaved by argonaute 2, therefore facilitating the stable association of miRNA sponges with ribonucleoprotein complexes loaded with the corresponding miRNA. Using these constructs, repression of miRNA targets was observed and proved effective in vitro silencing of miRNAs [69]. Theses effects were comparable with those obtained with 2′-O-methyl-modified oligonucleotides or LNA antisense oligonucleotides. Furthermore, sponges that contained only the heptameric seed were shown to effectively repress an entire miRNA family that shares by definition the same seed sequence [69].

### Blocking oncogenic MiRNAs using MiRNA masking antisense oligonucleotides

MiRNA masking effect strategy is designed to target single signalling cancer pathways. miR-mask (miRNA-masking antisense oligonucleotides) technology has been developed by Xiao and colleagues [70]. In contrast to miRNA sponges, miR-masks consist of single-stranded 2′-O-methyl-modified antisense oligonucleotides that are fully complementary to predicted miRNA binding sites in the 3′ UTR of the target mRNA [70]. In this way, the miR-mask covers up the miRNA-binding site to hide its target mRNA (Figure 5), thereby its effects are gene specific. This technology has been applied successfully in a zebrafish model to prevent the repressive actions of miR-430 in the transforming growth factor-β signalling pathway [71]. Although unwanted effects or off-target effects can be dramatically reduced with this approach, this may be a disadvantage for cancer therapy in which the targeting of multiple pathways may be desirable.

### Blocking oncogenic MiRNAs using inhibitors of oncogenic pathways

Several drugs may have the ability to modulate the expression of miRNAs by targeting signalling pathways that ultimately converge on the activation of transcription factors which in turn regulate miRNA encoding genes. Furthermore, it is possible to modulate the machinery that contributes to miRNA maturation and degradation processes. The identification of these compounds, however, is not straightforward and requires efficient screening of chemical libraries. Recently, Gumireddy and colleagues identified a method to screen for small-molecule inhibitors of miRNAs [72]. As a proof of concept for this approach, the investigators selected the frequently studied and up-regulated miRNA, miR-21. Complementary sequences to miR-21 were cloned into a luciferase reporter gene, which was then used as a sensor to detect the presence of specific mature miRNA molecules. The construct was transfected into HeLa cells, which express high miR-21 levels, resulting in low luciferase activity. Subsequently, a primary screen of more than several small-molecule compounds was done and an initial hit compound, diazobenzene 1, produced a 250% increase in the intensity of the luciferase signal relative to the untreated cells [72]. Additional characterization showed that this compound affects the transcription of miR-21. This strategy could be applied to the screening of small molecules as inhibitors for other distinct oncogenic miRNAs. These could be used with conventional cancer therapeutics to develop novel combined approaches for cancer treatment.

### Restoration of tumour-suppressor miRNAs

**Figure 6:**
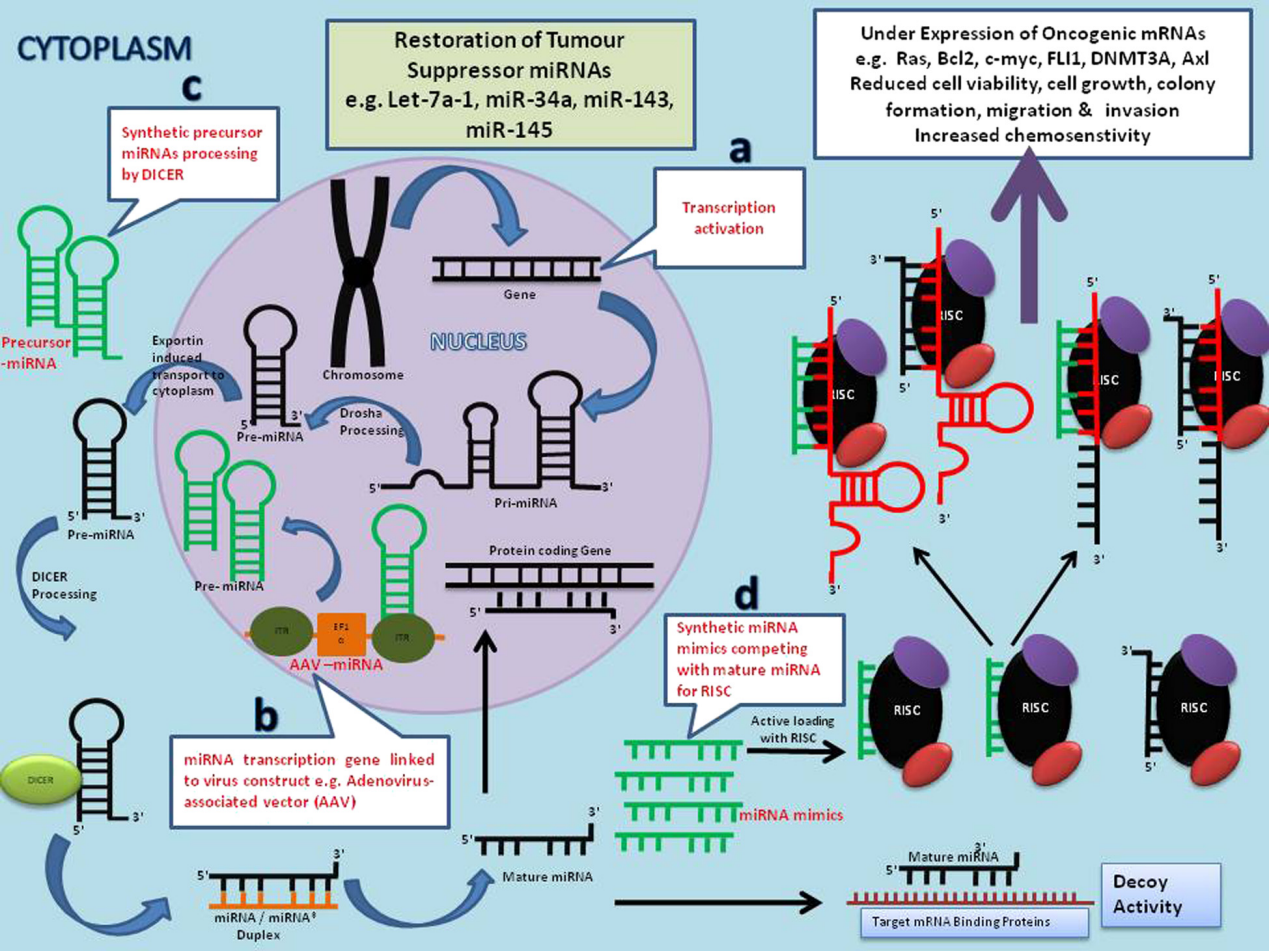
**Figure illustrates the mechanisms of restoration of tumour suppressor miRNAs.** The knockdown of oncogenic mRNAs can be achieved by restoration of tumour suppressor miRNAs by a) Transcription activation of miRNA transcribing gene b) Transfection of tumour cells with miRNA transcription gene linked to a virus construct e.g. adenovirus-associated vector (AAV) c) Introduction of synthetic precursor miRNAs in tumour cells d) Synthetic miRNA mimics competing with mature miRNAs for RISC.

The loss or downregulation of a tumour-suppressor miRNA could be overcome by introducing synthetic oligonucleotides i.e. mature miRNA mimics, miRNA precursors or pre-miRNA mimics into the CRC cells(Figure 6). Introduction of synthetic miRNAs with tumour-suppressor function in cancer cells have been shown to induce cell death and block cellular proliferation, transformation, invasion and migration in several studies as summarized in Table 4. The altered expression of tumour suppressor miRNAs have been studied in the context of cancer-associated transcription factors. P53 mutations have been found in 40–50% of CRCs. The p53 protein is a transcription factor that regulates multiple cellular processes in CRC development, either by regulating mRNA directly or by regulating miRNA indirectly. The absence of p53 mutations in adenomas suggests that loss of p53 is a critical step in progression of adenoma to carcinoma [73,74]. In addition, the miR-34 family has been strongly linked to p53 and loss of p53 has been linked to reduced levels of miR-34 in cancer cells. miR-34a restoration studies [75,76] have clearly demonstrated reduced cell survival, invasion and migration in CRC cell lines. In addition to a link with the p53 pathway, miR-34a encoding genes on their own have been identified as targets for the mutational or epigenetic inactivation in different cancers. Interestingly, miR-34a resides on the chromosomal locus 1p36, which has been proposed to harbor a tumor suppressor gene because it displays homozygous deletions in neuroblastoma and in other tumor types [77]. An unbiased screen for genes with tumor suppressive function on 1p36 also revealed miR-34a as a candidate tumor suppressor gene [78]. Therefore miR-34 targeted gene therapies hold a prime importance in the designing therapies for chemoprevention and to halt the tumour progression. miR-143 is another tumour suppressor miRNA, significantly found downregulated in CRC tissues (Table 1). The most significant study in this respect is restoration of miR-143 with miR-143 precursor resulting in reduced proliferation in SW480 colorectal cell lines and tumour suppression on xenografted tumors of DLD-1 human CRC cells [79,80]. Restoration of tumour suppressor miRNA by intravenous injection in mouse with liver cancer resulted in the suppression of tumorigenicity by reduced tumour growth and enhanced tumour apoptosis without signs of toxicity. This illustrates that tumour suppressor miRNA restoration strategy based gene therapy, if delivered efficiently to specific tissues may prove vital in future cancer treatments.

**Table 4:** Summary of studies involving the restoration of silenced miRNAs in CRC cell lines

**miRNAs**	**Cell Line**	**Target**	**Method**	**Reported effects of miRNA manipulation**	**References**
let-7a-1	DLD-1	Ras	let-7a-1 precursor	Reduced cell viability, cell growth and colony formation	Akao, et al, 2006 [27]
	SW480	c-myc			
miR-34a	DLD-1	Sirt1	miR-34a mimic	Reduced cell growth	Akao, et al, 2011[75]
		E2F3		Enhanced sensitivity to 5-FU	
miR-34a	Rko	Axl	pre-miR-34a	Reduced migration & invasion	Mudduluru, et al, 2011 [76]
miR-133b	SW-620 HT-29	tyrosine kinase MET	miR-133b precursor	Reduces cell proliferation Increased apoptosis	Hu, et al, 2010 [81]
miR-135a/b	CLY	CM-1 cytotoxicity	miR-135a,	Increased sensitivity to anticancer agent	Li , et al, 2011[82]
	HT-29		miR -135b mimics		
miR-137	SW1116,	Cdc42	miR-137 mimic	Reduces cell proliferation , Cell cycle arrest	Liu, et al, 2011[83]
	Lovo, Colo320				
				Inhibition of invasion	
miR-143	SW480,	*DNMT3A*	miR-143 precursor	Reduces cell proliferation	Ng, et al, 2009 [79]
	228				
miR-143	DLD-1		miR-143 precursor	tumor-suppressive effect on xenografted tumors of DLD-1 human CRC cells	Nakagawa, et al, 2010 [80]
miR-145	LS174T	FLI1	miR-145 precursor	Reduces cell proliferation and colony formation	Zhang , et al, 2011 [84]
	SW620 HCT116				
miR145	HCT116	IRS-1	ds-oligos miR145	Reduces cell proliferation	Shi , et al, 2007 [85]
	KO				
miR-185	SW1116 Lovo	RhoA Cdc42	hsa-miR-185 mimic	Cell cycle arrest & growth suppression	Liu, et al, 2010 [86]
miR-192	RKO, LoVo, DLD1, SW620	TYMS	Pre-miR-192	Reduces cell proliferation	Boni, et al, 2010 [87]
miR-195	HT29 and LoVo	Bcl-2	miR-195 mimic	Reduces cellular viability Increased apoptosis	Liu, et al, 2010 [88]
				Reduced colony formation	
miR-196a	SW480	HoxA7, HoxB8, HoxC8 HoxD8	miR-196a mimics	Reduced migration , invasion	Schimanski, et al, 2009 [89]
				Increased cellular adhesion	
				Increased sensitivity to platin derived anticancer agents	
miR-199a	Rko	Axl	pre-miR-199a	Reduced migration , invasion	Mudduluru, et al, 2011[76]
miR 200c	CLY	CM-1 cytotoxicity	miR 200c	Low chemosenstivity with high miR-200c	Li, et al, [82]
	HT-29		mimics		
miR-215	RKO, LoVo, DLD1, SW620	TYMS	pre-miR-215	Reduces cell proliferation	Boni, et al, 2010 [87]
miR-491	DLD-1	*Bcl-XL*	miR-491 precursor	Reduces cell proliferation	Nakano, et al, 2010 [90]
				Increased apoptosis	

### Modulation of miRNA processing

Alterations in miRNA processing machinery have also been implicated in cancer development and modulation of this machinery in part or in general can potentially lead to the discovery of new anticancer therapies. Researchers have investigated the global repression of miRNA maturation process in cells and have identified that the abrogation of miRNA processing pathway promotes the cellular transformation and tumorigenesis [91]. The inhibition of Dicer1 activity on its own has also been associated with cancer development, invasion and lymph node metastasis [91-94]. Therefore, speeding up the miRNA processing globally or by replacement of Dicer1 in cancer cells can alter their progression and invasive potential. However, the effects of alterations in miRNA biogenesis pathway have found to vary for different tumour types [92,93] and therapeutic strategy will vary dependent on the response for inhibition or acceleration of miRNA processing.

### Cancer stem cell directed miRNA therapy

Cancer stem cells or tumor-initiating cells have recently gained enormous attention. According to this hypothesis a subpopulation of cancer cells possesses unique characteristics of self renewal and multipotent differentiation; fundamental characteristics of embryonic and somatic stem cells [95]. As cancer stem cells or tumor-initiating cells are highly resistant to conventional chemotherapy and radiotherapy [96,97] there is further need for stem cell targeted therapy. Accumulating evidence indicates that miRNAs play functional roles in normal and cancer stem cell maintenance and differentiation. MiRNA expression signatures for differentiated cells are distinctly different from embryonic and somatic stem cells [98,99]. However, miRNA expressions in cancer stem cells have significant similarities with embryonic and somatic stem cells. Monzo and colleagues studied the common miRNAs for CRC and embryonic tissue and suggested that the miR-17-92 cluster and its target, E2F1, exhibit a similar pattern of expression in human colon development and colonic carcinogenesis [43]. Further studies dealing with miRNA biogenesis and stem cells; have suggested that mutation of the key proteins in miRNA biogenesis pathway fail to maintain the self-renewal and differentiation capacities [99-102]. Recent studies have demonstrated the role of let-7 in self renewal, differentiation and regulation of progenitor maintenance. Low expression of let-7 in cancer stem cells and restoration of stem cell related miRNAs expression level in cancer cells might prove pivotal in treating cancers by modulating cancer stem cells.

## Conclusions

In summary, there is strong evidence that miRNAs play a significant role in CRC development and progression. There is emerging evidence that miRNAs interact with different cancer signalling pathways and control cellular homeostasis. Changes in miRNAs expression levels results in alteration of this homeostasis and significantly contribute in cancer development and progression. Cancer specific miRNAs are detectable in body fluids and can potentially be used as novel biomarkers for CRC detection and prediction of cancer specific survival. MiRNAs influence oncogenic potential and therefore strategies to manipulate oncogenic or tumour suppressor miRNAs can successfully halt tumour progression. Furthermore, miRNA manipulation strategies can potentially be used as an adjuvant to other forms of anticancer therapies as miRNA manipulation can be used to sensitise drug resistant tumours. Finally, stem cell directed miRNA based therapies can be used to control stemness and latency of cancer stem cells in order to prevent the recurrence of tumour. However, miRNA based gene therapy is still in its infancy, but does hold great potential to replace current anticancer therapies. Further work is needed on an efficient, tissue specific delivery system and strategies to avoid side effects.

## Competing interest

The authors declare that they have no competing interests.

## Authors’ contribution

MIA: literature search, review of studies, critical analysis of studies, layout of manuscript, design of figures and tables. MP: literature search, review of studies, critical analysis of studies. JHP: critical review of manuscript and analysis of the quality of manuscript. BS:, reviewed the quality of manuscript. JSJ: Review of quality , structure and design of manuscript. All authors read and approved the manuscript.
